# Interleukin-6 and Lymphocyte Count Associated and Predicted the Progression of Frailty Syndrome in Prostate Cancer Patients Undergoing Antiandrogen Therapy

**DOI:** 10.3390/cancers12071716

**Published:** 2020-06-29

**Authors:** Cristina Buigues, Rut Navarro-Martínez, Vanessa Sánchez-Martínez, María Serrano-Carrascosa, José Rubio-Briones, Omar Cauli

**Affiliations:** 1 Department of Medicine and Nursing. University of Valencia, 46010 Valencia, Spain; cristina.buigues@uv.es (C.B.); Rut.Navarro@uv.es (R.N.-M.); Vanessa.sanchez@uv.es (V.S.-M.); 2 Frailty Research organized Group (FROG), University of Valencia, 46010 Valencia, Spain; 3 Department of Haematology, Hospital General Universitario, 46014 Valencia, Spain; 4 Department of Urology, Fundación IVO, 46009 Valencia, Spain; marietadelao77@gmail.com (M.S.-C.); jrubio@fivo.org (J.R.-B.)

**Keywords:** interleukin-6, interleukin-1 beta, inflammation, leukocytes, biomarker, geriatric assessment

## Abstract

Frailty syndrome is a functional state that includes a loss of ability to react to stressors, and is associated with poor outcomes, morbidity and premature mortality. The first line treatment in many men with prostate cancer (PCa) consists of an androgen-deprivation therapy (ADT) which can promote or favor frailty syndrome and ADT may therefore favor the progression of frailty over time. Among the pathophysiological bases of frailty, the presence of chronic low-grade inflammation has been associated with its adverse outcomes, but longitudinal studies are needed to validate these biomarkers. In this study, we prospectively evaluate frailty syndrome and blood inflammatory markers (IL1-beta, IL-6, IL-8, TNF alpha, C reactive protein) and leukocytes were measured at baseline and an average of 1 year later in PCa under ADT. Frailty was defined as having three or more of the following components: low lean mass, weakness, self-reported exhaustion, low activity level, and slow walking speed; prefrailty was defined as having one or two of those components. Multinomial regression analysis showed that among the inflammatory biomarkers, those significantly and repeatedly (baseline and follow-up time points) (*p* < 0.05) associated with frailty syndrome were high IL-6 levels and low lymphocyte counts in blood. Other biomarkers such as IL-8, monocyte counts and C reactive protein were significantly associated with frailty syndrome (*p* < 0.05) in cross-sectional analyses, but they do not predict frailty progression at 1 year-follow-up. Receiver operating characteristic curve analysis showed that both lymphocyte counts and IL-6 concentration significantly (*p* < 0.05) (although moderately) discriminate PCa patients that progressed in the severity of frailty syndrome. IL-6 and lymphocytes count are possible biomarkers, useful for identifying frail patients and predicting the progression of frailty in PCa under ADT. Our study suggests the use of these biomarkers to guide clinical decisions on prostate cancer treatment based on a multidisciplinary approach.

## 1. Introduction

Prostate cancer (PCa) is the third most common type of tumor in Europe, and the third most frequent cause of death from cancer in men [1]. Treatment selection is based on the disease risk category, the patient’s age and comorbidities, the patient’s preferences and the adverse effects profile of the treatment [2]. PCa is an androgen-dependent disease. Hormone therapy, also called androgen deprivation therapy (ADT) with luteinizing hormone-releasing hormone (LHRH) analogues or antagonists is the most widely used therapeutic modality in advanced or metastatic and hormone-sensitive PCa [3]. LHRH analogues are more commonly used, and have replaced surgical castration as the standard of care in ADT, because these agents have potential for reversibility and prevent the physical and psychological discomfort associated with orchiectomy [4,5]. The ADT is commonly achieved by administering LHRH agonists is in the first line of treatment of castration-sensitive PCa [4].

However, ADT is associated with a number of adverse effects that could limit their use over time in clinical settings. These include reduced muscle mass or sarcopenia, muscle weakness, a decline in bone mineral density, increased fat mass, increased insulin resistance, reduced activity levels, increased risk of falls and fractures, increased risk of depression and fatigue [6]. In men with PCa, ADT therefore contributes to functional change and may increase rates of frailty and its adverse consequences [7,8]. Age-related decline in serum testosterone has been suggested to be associated with individual components of frailty such as diminished energy, muscle strength and physical function [9,10,11,12]. For these reasons, it is crucial to detect frail patients before deciding on the most suitable treatment [13]. Frailty is a state of high vulnerability to adverse health outcomes, including disability, dependency, falls, long-term care needs and mortality [14]. One of the most widely accepted methods for measuring frailty is the one developed by Fried et al. [15]. This approach defines frailty as a clinical syndrome in which three or more of the following parameters are present: unintentional weight loss, self-reported psychophysical exhaustion, decreased grip strength, slow walking speed, and low physical activity [14,15]. This clinical syndrome closely overlaps with the known toxicities of ADT, and could therefore lead to the appearance of frailty in non-frail individuals, or accelerate the progression of frailty in individuals with an already established level of frailty [8,16,17]. 

To date, no studies have evaluated if ADT in PCa patients associates with the progression of frailty syndrome over time, taking into account that ADT in PCa greatly reduce the levels of circulating testosterone sometimes achieving undetectable levels, in order to limit cancer relapses and disease progression [18,19,20] and could accelerate the progression of frailty syndrome. There is a consistent body of evidence that relate testosterone deficiency to systemic inflammatory responses e.g., both animal studies and human studies showed that testosterone deficiency is associated with an increase in pro-inflammatory cytokines and testosterone substitution reduced pro-inflammatory cytokines in patients with coronary artery disease, prostate cancer and diabetes mellitus through the decrease in pro-inflammatory cytokines (IL-1β, IL-6, and TNF-alpha) [21,22].

Immune system alterations, impacting both adaptive and innate immune responses, have emerged as one of the most relevant “hallmarks of aging” processes and immunological factors were among the biomarkers related to the pathophysiology of frailty syndrome [23]. The role of systemic inflammation as a key element of functional decline during aging is based on the hypothesis that the aging process is related to a systemic increase in pro-inflammatory mediators from various sources [24,25]. The increase in circulating pro-inflammatory cytokines (IL-6, TNF-alpha, IL-beta) and other inflammatory markers (PCR and white blood cell counts) concentrations have been also associated with frailty syndrome in several studies [26,27,28,29,30,31]. Cytokines overproduction such as IL-6, TNF-alpha, IL-8 have been also involved in the PCa pathophysiology [32,33,34,35,36,37] and thus inflammation could be common pathophysiological framework for frailty syndrome and ADT treatment in PCa patients.

Recent international guidelines state that in order to optimize treatments for older cancer patients, it is necessary to include geriatric assessments [4,17,38]. Among them, the screening for frailty syndrome is useful for determining the appropriateness of treatments or triggering a referral to a geriatrics team [39].

The identification of biomarkers associated with the progression of frailty syndrome can help understand the mechanisms of frailty, and facilitate the diagnosis and development of interventions to delay or reverse its progression. Considering that frailty syndrome is a potentially reversible state, a better understanding of the characteristics associated with the improvement or maintenance of the state of frailty in some subjects makes it possible to determine the early risk factors and could enable identification of individuals who are more likely to benefit from specific interventions to reduce or prevent frailty syndrome. In this study, we pursued three main objectives: (1) to analyze the progression (1-year follow-up) of frailty syndrome and its components in patients with PCa undergoing ADT; (2) to evaluate the most reproducible changes in peripheral inflammatory markers associated with the severity of frailty syndrome by comparing the results obtained at two cross-sectional levels e.g., at baseline and at follow-up; (3) to analyze if these biomarkers at baseline can predict the progression of frailty syndrome. 

## 2. Results

### 2.1. Characteristics of the Study Population

Only 39 of the 46 men recruited at baseline (who were undergoing ADT at least six months prior to joining the study because of their disease progression or initial metastatic stage) were evaluated in the one-year follow-up study. The time of evaluation for the follow-up was 10.8 ± 2.6 months. The drop-outs were due to progression of prostate cancer and the requirements of other pharmacological treatments e.g., five patients became resistant to castration and two metastatic patients switched to chemotherapy treatment. Their ages ranged from 51 to 92 years, with a mean age of 71.9 (± 9.8). As shown in Table 1, almost all the participants were married (*n* = 34; 87.2%) and only 10.3% lived alone. As regards their educational level, most patients reported having completed school, and 15.2% of those (*n* = 7) reported having completed university studies. Most patients (66.7%) had undergone radical prostatectomy. The pharmacological treatment with LHRH analogues consisted of depot administration of leuprorelin (*n* = 10 patients) or triptorelin (*n* = 29 patients). 

### 2.2. Evaluation of Frailty Syndrome in the Study Sample

**Figure 1:**
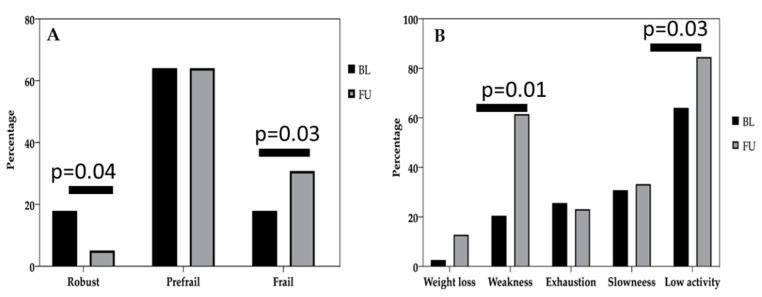
Evaluation of frailty syndrome in the study sample. Frailty was measured according to the 5 criteria proposed by Fried et al. [15]. (**A**) Percentage of the categories of frailty expressed as follows: participants who met 3 or more criteria were classified as frail, those who met 1 or 2 were prefrail, and those who did not meet any of the criteria were non-frail (robust). (**B**) Number of frailty criteria met in the sample, expressed as a percentage of the entire population. BL: Baseline; FU: Follow-up.

The percentages of frailty phenotype at baseline and at follow-up are shown in Figure 1. We analyzed each of the five Fried criteria at follow-up (FU), and compared them to the percentage of the same criteria at baseline (Figure 1). The most frequent criterion in the overall sample was low physical activity (64.1% vs. 84.6%), followed by weakness or low muscle strength (20.5% vs. 61.5%), slow gait speed (30.8% vs. 33.3%), self-reported fatigue (25.6% vs. 23.1%), and finally, involuntary weight loss in the last year (2.6% vs. 12.8%). When categorizing the frailty criteria according to Fried’s physical phenotype there was a significant (*p* < 0.05) reduction in the percentage of robust (non-frail) individuals and a significant (*p* < 0.05) increase in the percentage of frail individuals at follow-up. Based on the Fried criteria (Fried, 2001) 30.8% of the subjects were classified as frail (met ≥3 frailty criteria), 65.2% as prefrail (met 1 or 2 frailty criteria), and the remaining 17.4% as robust or non-frail (not meeting any of the frailty criteria). At follow-up, 59% of patients had a worse level of frailty syndrome compared to baseline (as they met at least one more frailty criterion at the follow-up visit), whereas 41% of patients improved (fewer frailty criteria met at follow-up compared to the baseline) or remained the same (the same number of frailty criteria met at baseline and at follow-up). 

When we analyzed each of the 5 criteria between baseline and follow up, there were significant differences between physical activity as a function of time and intensity (M 223.33 SD ± 323.42 minutes/week vs. M 76 SD ± 210.09 minutes/week, *p* = 0.005, Wilcoxon test); slow walking speed (5.18 seconds ±1.16 vs. 5.62 seconds ± 1.36, *p* = 0.015, Wilcoxon test) and low muscle strength (M 35.12 Kg ± SD 8.19 vs. M 27.15 ± 9.69 Kg, *p* < 0.001, Wilcoxon test). However, despite the significant reduction in walking speed at follow-up compared to the baseline, the percentage of subjects that met the “slowness” frailty criterion was similar at baseline and follow-up. There were no significant differences between involuntary weight loss (*p* = 0.807, Wilcoxon test) and self-reported fatigue (*p* = 0.701, Wilcoxon test).

At baseline, the only sociodemographic variable that was significantly associated with frailty syndrome was age (*p* = 0.009, Kruskal–Wallis test), with older patients being more frail than younger patients. There were no significant differences between the frailty category distribution (robust, prefrail and frail) as regards socio demographic variables such as age (*p* = 0.10, Kruskal–Wallis test), marital status (*p* = 0.11, Chi-squared test), educational level (*p* = 0.38, Chi-squared test), Barthel score (*p* = 0.90, Kruskal–Wallis)and Charlson Comorbidity Index (*p* = 0.44, Kruskal–Wallis). No significant differences were found between the level of frailty and clinical variables related to PCa such as the National Comprehensive Cancer Network (NCCN) risk score (*p* = 0.28, Chi-squared test), Gleason score (*p* = 0.60, Kruskal–Wallis test) or the presence or absence of bone metastasis (*p* = 0.71, Chi-squared test), having undergone a prostatectomy or otherwise (*p* = 0.21, Chi-squared test), BMI (*p* = 0.29 Kruskal–Wallis test) or the type of LHRH analogue administered (*p* = 0.48, Chi-squared test).

At follow-up, there were significant differences in the Charlson Comorbidity Index (*p* = 0.002, Kruskal–Wallis) between robust, prefrail and frail individuals. There were no significant differences between frailty categories in the frailty category distribution (robust, prefrail and frail) and socio-demographic variables such as age (*p* = 0.28, Kruskal–Wallis test), marital status (*p* = 0.97, Chi-squared test), and educational level (*p* = 0.21, Chi-squared test), Barthel score (*p* = 0.43, Kruskal–Wallis). No significant differences were found between the level of frailty and clinical variables related to PCa, such as the Gleason score (*p* = 0.40, Kruskal–Wallis test) or the presence or absence of bone metastasis (*p* = 0.20, Chi-squared test), having undergone a prostatectomy or otherwise (*p* = 0.26,Chi-squared test), BMI (*p* = 0.53, Kruskal–Wallis test) or the type of LHRH analogue administered (*p* = 0.66,Chi-squared test).

### 2.3. Association Between Inflammatory Markers and Frailty Syndrome

**Figure 2:**
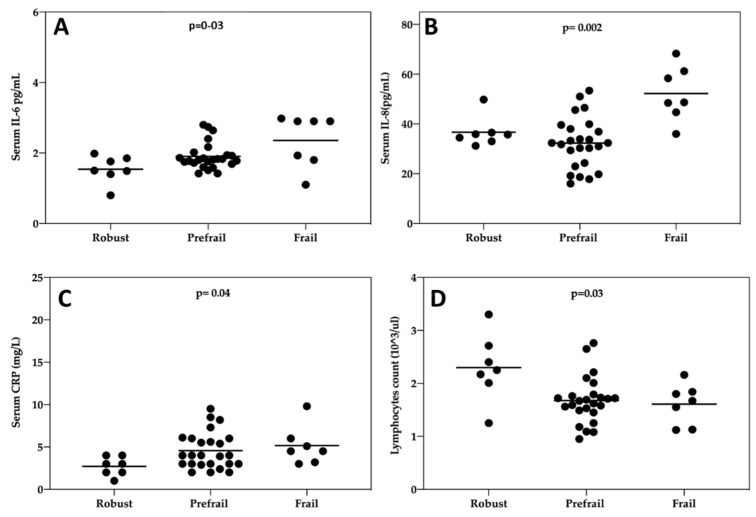
Evaluation of relationship between frailty syndrome and inflammatory markers at baseline. Frailty was measured according to the 5 criteria proposed by Fried et al. (see Methods). (**A**) IL-6 (pg/mL), (**B**) IL-8, (pg/mL) (**C**) CRP (mg/L), (**D**) lymphocytes s(1 × 10^3^/µL) were measured in the blood and were plotted against the categories of frailty.

At baseline, there were significant differences in IL-6 (*p* = 0.03, Kruskal–Wallis test), IL-8 (*p* = 0.002, Kruskal–Wallis test) and PCR (*p* = 0.04, Kruskal–Wallis test) concentration in blood between robust, prefrail and frail individuals (Figure 2A–C). Among the leukocyte subtypes, the lymphocyte count differed significantly (*p* = 0.03) between different levels of frailty (Figure 2D). At baseline, the other inflammatory markers or leukocyte subtypes were not significantly associated with frailty syndrome (*p* > 0.05 in all cases). In the multinomial logistic regression analyses, a higher IL-6 was associated with a significantly increased OR of being frail (OR = 169.5, 95% CI = 3.0−9691.6; *p* = 0.013), as was a higher IL-8 (OR = 1.2, 95% CI = 1.04−1.44; *p* = 0.014) compared to being non-frail. A higher lymphocyte count was associated with a significant OR of being frail (OR = 0.11, 95% CI = 0.001−0.75; *p* = 0.036. In the regression model, at baseline, an increase in PCR concentration did not significantly increase the OR of being frail (*p* = 0.18). 

At follow-up, there were significant differences between robust, prefrail and frail individuals for IL-6 (*p* < 0.001, Kruskal–Wallis test), and the monocyte count (*p* = 0.04, Kruskal–Wallis test). At follow-up, the other inflammatory markers and leukocyte subtypes were not significantly associated with frailty syndrome (*p* > 0.05 in all cases). In the multinomial logistic regression analyses, a higher IL-6 was associated with a significantly increased OR of being frail (OR = 96.8, 95% CI = 2.6−3559.9; *p* = 0.011). In the regression model, at follow-up, an increase in monocytes count did not significantly increase the OR of being frail (*p* = 0.18). 

**Figure 3:**
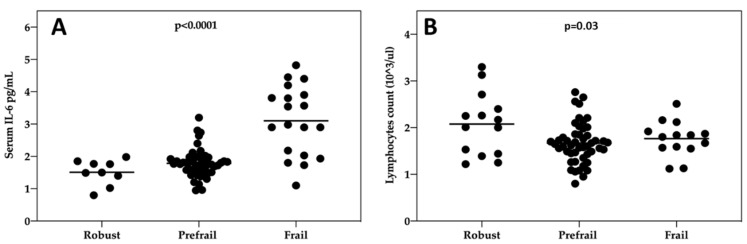
Evaluation of the relationship between frailty syndrome and IL-6 and lymphocytes count at baseline and follow-up pooled together. Frailty was measured according to the 5 criteria proposed by Fried et al. (2001) [15]. (**A**) IL-6 (pg/mL), (**B**) lymphocytes count (1 × 10^3^/mL) were measured in the blood, and plotted against the categories of frailty, which was expressed as follows: participants who met 3 or more criteria were classified as frail, those who met 1 or 2 were prefrail, and those who did not meet any of the criteria were nonfrail (robust).

When we pooled together data of the two biomarkers that significantly associated with the severity of frailty syndrome both at baseline and follow-up e.g., IL-6 (Figure 3A) and lymphocytes counts (Figure 3B), we observed a strong significant effect with IL-6 levels (*p* < 0.0001. Kruskal–Wallis test) and a milder significant effect for lymphocytes counts (*p* = 0.03, Kruskal–Wallis test)

**Figure 4:**
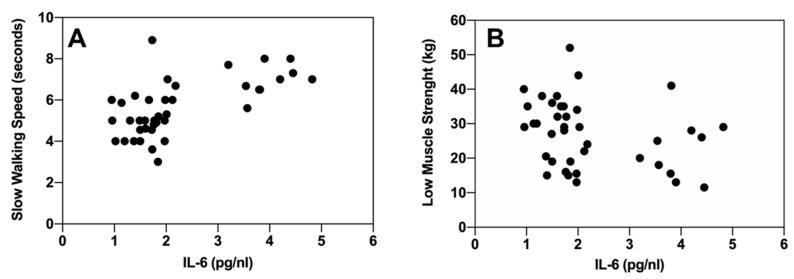
Correlation between IL-6 and individual criteria of frailty syndrome at follow-up. Frailty was measured according to the Fried criteria [15]. (**A**) IL-6 (pg/mL) and slow walking speed and (**B**) IL-6 (pg/mL) and low muscle strength.

Since the only socio-demographic variable positively associated with a severe frailty level was age, we evaluated whether age correlates with any of the inflammatory biomarkers. No significant correlations were found between age and lymphocytes, monocytes, cytokines (IL-6, IL-1beta, IL-8, TNF-alpha), or PCR (*p* > 0.05 in all cases). Next, we evaluated the correlation between individual criteria of frailty syndrome at follow-up and monocytes count and IL-6 at follow-up. There was a significant inverse correlation between the monocyte count and physical activity (Rho = −0.39, *p* = 0.01; Spearman test). Serum IL-6 was associated with a slower walking speed (Rho = 0.66, *p* < 0.001; Spearman test) (Figure 4A), self-reported fatigue (Rho = 0.37, *p* = 0.02; Spearman test) (Figure 4B), and inversely correlated with weakness and low muscle strength (Rho = −0.35, *p* = 0.02; Spearman test). Nonetheless, IL-6 was not significantly associated with involuntary weight loss (Rho = −0.11, *p* = 0.50; Spearman test) or physical activity (Rho = −0.21, *p* = 0.19; Spearman test).

No significant correlations were found for proinflammatory cytokine IL-beta, TNF-a, IL-8, or the inflammatory markers fibrinogen and CRP (*p* > 0.05 in all cases). Likewise, there were no statistically significant differences in IL-6, IL-beta and monocyte count between patients with or without metastases (IL-6 *p* = 0.149; IL-beta *p* = 0.402; monocytes *p* = 0.307) or prostatectomy (IL-6 *p* = 0.770; IL-beta *p* = 0.930; monocytes *p* = 0.370). 

### 2.4. Analysis of Clinical Variables and Inflammatory Markers as Predictors of Progression of Frailty Syndrome

In order to better understand the second outcome of the study e.g., which inflammatory biomarker can predict the progression of frailty syndrome at 1 year follow-up, all the biomarkers were analyzed as dichotomic events for frailty progression (progression or not progression, stable or improved level of frailty) (Table 2). There was a significant difference in the baseline lymphocytes counts (*p* = 0.022) and IL-6 concentration (*p* = 0.038) in men who progressed in frailty syndrome severity compared to those who did not progress at the one-year follow-up period (Table 2). No significant differences were observed for other hematological markers or inflammatory cytokines. 

In the multinomial logistic regression analyses with all inflammatory biomarkers associated with frailty at baseline, the significant effects were observed for IL-6 and lymphocytes counts both of which predicted the progression of frailty syndrome at follow-up. A higher log (IL-6) was associated with a significantly increased risk of frailty progression at 1 year follow-up (hazard ratio (HR) = 2.2, 95%CI = 1.23–6.39.8; *p* < 0.05), as was a lower lymphocytes count (HR = 1.2, 95%CI = 1.06–1.45; *p* < 0.05). In contrast, no significant differences were observed for the remaining inflammatory biomarkers and clinical variables for the risk of frailty regression (*p* > 0.05 in all cases). 

Next, we performed a receiver operating characteristic (ROC) as a useful tool for evaluating the diagnostic power of these biomarkers. This analysis provided an exhaustive look at the trend of sensitivity over all cutoffs, and thus provided information about the relationship between the sensitivity and the specificity of IL-6 and lymphocytes count.

**Figure 5:**
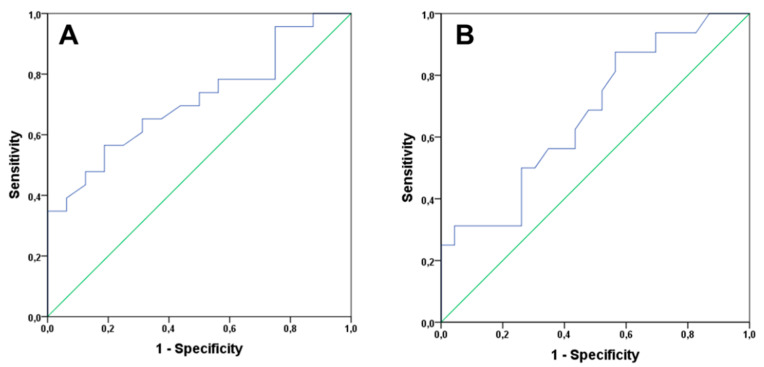
Receiver operating characteristic (ROC) curve for lymphocyte count (**A**) and IL-6 concentration (**B**) and the ability to discriminate between patients who progressed or otherwise in frailty syndrome.

For the lymphocyte count, the area under the curve value was 0.713 with CI 95% 0.553–0.873 (Figure 5A) with acceptable values, and the cut-off value of 1.7 × (10^3^/µL) lymphocyte count has a sensitivity of 65.2% and a specificity of 68.7%. For IL-6, the area under the curve value was 0.674 with CI 95% 0.503–0.845 (Figure 5B), and the cut-off value 1.79 pg/mL has a sensitivity of 69.0% and a specificity of 52.8%.

## 3. Discussion

The evaluation of biomarkers associated with and predictive of frailty syndrome is an interesting field for a multidisciplinary approach to older cancer patients which enables a diagnosis of frailty, evaluation of the side-effects of cancer treatments, and monitoring of the intervention to prevent or reduce the progression of frailty syndrome [26,40,41,42]. The reduction of androgens in PCa patients undergoing ADT could promote the progression of frailty, as reduced testosterone levels have been associated with frailty [9,12] and it can synergize with other mechanisms linked to frailty, such as low-grade inflammation as mechanisms to accelerate frailty progression. In addition, androgens have a strong and complex modulatory role in the production of inflammatory cells and cytokines [21,43,44].

Several phenomena overlap in this longitudinal study involving a population of men with PCa undergoing ADT, immunosenescence and “inflammaging”, as a potential consequence of ADT and progression of frailty syndrome. It is therefore important to understand the directionality of the inflammation and frailty relationships, and to identify the biomarkers that predict the progression of frailty over time.

Several studies have suggested that elevations in the levels of circulating proinflammatory cytokines and their receptors, such as tumor necrosis factor alpha (TNF-α), interleukin (IL)-6, interleukin (IL)-1 receptor antagonist (IL-1Ra) and soluble TNF receptors are strong independent risk factors of morbidity and mortality in the elderly [45,46,47,48]. Furthermore, some of these inflammatory markers have been also suggested as playing an important role in the onset and progression of prostate cancer (PCa) [49,50]. Low testosterone levels have also been associated with frailty [9,12]. This entire body of evidence suggests that frailty could be accelerated during the treatment of PCa patients with ADT. The identification of biomarkers that could help clinicians to identify frail PCa patients will help to tailor appropriate clinical decisions, and limit adverse outcomes related to frailty [8,26].

Our results show that some inflammatory markers, such as monocyte counts, blood IL-8, IL-1 beta, and PCR concentration, are related to the pathophysiological evolution of PCa patients undergoing ADT treatment, and were associated with frailty syndrome at baseline or at follow-up. The association is particularly linked to some alterations in blood cell types such as an increase in monocyte count, which is significantly correlated with reduced physical activity criterion of frailty. Chronic low-grade inflammation-induced expansion of the myeloid cell lineage in the frail population is consistent with previous results [51,52]. Mouse models have revealed a role for myelomonocytic cells in driving malignant progression and metastasis [53,54]. These models have also shown that as precursors of tumor-associated macrophages, inflammatory monocytes contribute to tumor metastasis and immunosuppression [55]. Among the inflammatory markers evaluated in our study, those repeatedly associated with frailty syndrome at both baseline and follow-up were reduced lymphocyte counts and high IL-6 levels. These results obtained in PCa patients confirm and extend the observations which correlated frailty syndrome to chronic low-grade inflammation in several populations and involved both the innate and adaptive components of the immune system [56,57,58] and in this study, we reported the role of some inflammatory biomarker confirmed in longitudinal studies which validated some of the biomarkers associated with frailty syndrome in cross-sectional studies. Longitudinal designs for biomarker traits are very interesting for a number of reasons. First, collecting repeated biomarker measurements over time allows investigators to characterize both the overall and individual trajectories in these measurements. This helps investigators to understand the overall effects, as well as how individuals vary around these average effects. Distinguishing individual heterogeneity from the average trend is impossible without longitudinal data. This distinction is important for understanding individual between-subject variation, as well as in developing predictors of disease outcome from longitudinal marker data.

These inflammatory biomarkers (IL-6 and lymphocytes) were particularly strongly associated with some frailty criteria e.g., grip strength, slower walking speed and self-reported fatigue, confirming their role in a proper neuromuscular function [59,60]. Enhanced chronic inflammation, reflected in an increased level of pro-inflammatory cytokines, is related to muscle weakness in the upper and lower extremities, which manifests as the frailty indicators of reduced handgrip strength and slow gait speed [56,61,62].

Our study demonstrated for the first time that IL6 levels can help to classify PCa patients whose frailty syndrome severity increased during ADT treatment, i.e., the area under the ROC curve obtained for IL-6 (0.67) indicates a moderate accuracy in the predictive value of this biomarker for frailty. At concentrations >1.79 pg/mL, frail subjects can be identified with moderate accuracy. Furthermore, other reports suggest that IL-6 may be one of the key factors contributing to increased tumoral growth in the absence of androgens and as such, IL-6 could be an oncogenic factor in PCa and a regulator of prostate cancer progression [32,50]. The identification of pathways leading to the expansion of stem-like cancer cells could therefore provide innovative strategies for targeting this cell subpopulation and preventing disease progression and recurrence. PCa treatment in advanced stages may be successful if appropriately combined with other established or experimental treatments [50].

Low lymphocyte counts predicted the progression with frailty and this effect has been linked to a deterioration of muscular activity, which is traditionally involved in the physiopathology of frailty syndrome [63,64]. Several studies support the association between lymphopenia and inflammation/immunosuppression, which are both crucial mechanisms linked to frailty [65,66]. A recent observational prospective single-center study in patients with coronary disease showed that in multivariate analysis, lymphocyte counts were inversely related to the odds of frailty, with an exponential increase in risk [66].

In our results the majority of patients had overweight or obesity. Although there was a loss of body weight during the follow-up, we cannot state whether the change was due to loss of fat or/and lean mass, and future studies about the role of sarcopenic obesity in PCa patients under ADT and their relationship with frailty syndrome are warranted. Several studies indicate that excessive adiposity contributes to physical frailty and functional limitations at advanced ages [67,68]. Body composition is altered significantly by ADT, and, as a consequence of beginning the treatment, men lose lean muscle mass while gaining fat mass [69]. Adipose tissue is metabolically active, and promotes systemic inflammation and oxidative stress [70]. In addition, obesity exacerbates fat infiltration into muscles (i.e., myosteatosis), which in turn, contributes to muscle dysfunction and physical frailty [71].

Better information on the epidemiology of frailty in prostate cancer patients is essential in a global approach to cancer care for older people. It will guide shared treatment decisions based on an individualized balance of risk and benefits [72]. Our findings support routine assessment of individual frailty and fitness in older cancer patients to guide treatment decisions. Failure to detect frailty potentially exposes older cancer patients to treatments from which they might not benefit, and indeed they may be harmed [72]. 

This study has some limitations. First, the sample size of our study is small, so it did not allow us to identify differences in the progression and accuracy of frailty between patients with or without certain clinical features, such as the presence of metastases or with the effect of prostatectomy. Second, because age is associated with low levels of inflammation and frailty, it could be interesting to include more middle-aged individuals and to compare the outcomes between older (>65 years old) and younger patients. We did not evaluate the type of lymphocyte cells involved in these effects, and we do not know which cell types are involved in higher releases of IL-6, but prostate tissue or the spread of tumor in bones seems not to be involved, since no differences for the presence of prostatectomy or bone metastasis in these conditions were found. 

In conclusion, our findings suggest that testing frailty syndrome, especially in those PCa patients with higher IL-6 and reduced lymphocyte counts, could be an important predictor in clinical decisions regarding frailty and PCa interventions under ADT. 

## 4. Materials and Methods

This is a prospective longitudinal observational study, which was performed between January 2019 and March 2020. Thirty-nine patients at the Urology Service of the Valencia Oncology Institute (IVO) diagnosed with prostate cancer and treated with continuous ADT treatment were recruited when they attended the follow-up appointment with the specialist.

The inclusion criteria were a diagnosis of PCa (all stages) and the prescription of ADT. The exclusion criteria for all groups were severe cognitive impairment (Mini-Mental State Examination (MMSE) score <21), severe psychiatric disorders or blindness, and acute infections. The trial was carried out in compliance with the guidelines of the Declaration of Helsinki, and the study protocol was approved by the local Ethics Committee (University of Valencia, Reference number: H1511682610849). All participants gave written informed consent before being enrolled in the study.

A questionnaire was administered to determine the sociodemographic characteristics of the men in the study (marital status, educational level, employment status, cohabitation, age, body mass index, smoking status). The clinical variables of prostate cancer were obtained from the patients’ medical records, and were the basis for recording the following parameters: date of cancer diagnosis, clinical stage at the time of diagnosis, type of androgen deprivation treatment, time since the last administration of the treatment, previous prostatectomy and presence of bone metastases.

To assess frailty, a questionnaire was developed according to the 5 Fried criteria of frailty: involuntary weight loss, chronic self-reported fatigue, weakness/grip strength, walking speed and physical activity [15]. The criteria were assessed as follows: (1) involuntary weight loss was recorded by listing the patient’s usual and current weight, height, and BMI and by completing the closed question “Have you lost weight in the last year?” This weight loss was defined as an unintentional loss of 4.5 kg or more in the past year; (2) chronic self-reported fatigue was obtained by asking the patient, "How often a week do you feel tired in everything you do?" The response options were: never, sometimes (1–2 times a week), often (3–4 times a week), and always (almost every day). This criterion was considered present if the patient answered the question with "often" or "most of the time" on the Center for Epidemiologic Studies-Depression scale [73]; (3) to measure muscle weakness, grip strength (Kg) was measured taking the recording of the palmar grip strength obtained with a hydraulic dynamometer as a benchmark (Jaymar, J.A. Preston, Corp., Jackson, MS, USA). Three consecutive measurements of muscle strength were performed in each hand to do this. The arms were alternated, leaving a muscle recovery time of approximately one minute, and the highest value of the three measurements made was recorded, evaluated in accordance with the standards for Hispanic Established Populations in Epidemiological Studies of the Elderly [74]; (4) to assess gait speed, we calculated the time it took the patient to travel 4.6 meters at their usual walking speed. To do so, our values were as follows: men taller than 173 cm: ≥6 seconds, height < 173 cm ≥7 seconds. The patient was considered to have a slow walking speed when the speed was in the worst quintile for the group, after adjusting for sex and height according to the parameters of the short physical performance battery [75]; (5) the International Physical Activity Questionnaire (IPAQ) validated in Spanish was used, which assesses physical activity as a function of time and intensity to measure low physical activity. The total amount of energy spent on activities in 1 week was calculated and divided into quintiles. The individuals in the lowest quintile (20.0% less active) received a positive score for this frailty criterion.

According to the Fried criteria [11], participants were considered frail if they met at least 3 criteria, and prefrail if they met 1 or 2. All the measurements were performed by trained members of the Department of Nursing at the University of Valencia, using a questionnaire with detailed instructions.

### 4.1. Measurement of Inflammatory Markers

Proinflammatory cytokine serum TNF-a and IL-6 were measured using commercial enzyme-linked immunosorbent assay kits, according to the manufacturer’s instructions (TNF-a (ab100654), IL-6 (ab46042), IL-1 beta (ab46042), IL-8 (ab46032) Human ELISA Kit, Abcam). We also counted the five-part white blood cell differential and acute phase reactant proteins such as fibrinogen and CRP, in order to better characterize the analysis of inflammatory biomarkers.

### 4.2. Measurement of Hematological and Biochemical Markers

Blood serum (5 mL) was acquired by taking blood with BD Vacutainer tubes and centrifuging them at 500 *g* for 10 minutes at room temperature to separate the blood components. All samples were kept at 4 °C to 6 °C, and processed within 2 hours of collection. Hemoglobin concentration and leukocyte, erythrocyte and platelet counts were obtained with different automated instruments in hospital hematology laboratories. Biochemical and hematological analyses were also performed. 

### 4.3. Sociodemographic and Clinical Variables

The variables included sociodemographic characteristics (age, marital status, body mass index (BMI)), clinical variables of PCa (clinical stage at diagnosis, time since diagnosis, type of drug for ADT, previous prostatectomy, and bone metastases). We measured 5 frailty criteria (involuntary weight loss, low energy or exhaustion, slow mobility, muscle weakness, and low physical activity) according to Fried et al. [9]. We assessed the tumor PCa stage according to NCCN risk stratification. Geriatric assessment was based on the Barthel index for basic activities of daily living, and the Charlson Comorbidity Index adjusted for age.

### 4.4. Statistical Analysis

The continuous variables were expressed as the mean and standard deviation, and the categorical variables as the absolute value with their percentage. The normal distribution of each variable was assessed with the Shapiro–Wilk test in order to determine whether a parametric or non-parametric test should be applied. The correlation between quantitative variables was determined by Spearman’s (for non-normal data distribution) or Pearson’s (for normal data distribution) correlation test. The differences between the two groups were analyzed with the non-parametric Mann–Whitney U test or the parametric Student t test. The differences between the three groups were analyzed using the non-parametric Kruskal–Wallis test or parametric analysis of variance (ANOVA), followed by post hoc testing where appropriate. We evaluated linear correlations between continuous variables using the Spearman’s rank test. Bivariate analysis was conducted between data recorded at baseline and at follow-up. Multinomial logistic regression models were used to assess the effects of the variables found to be significant in the bivariate analysis for the risk of being frail or prefrail vs. non-frail. Logs (of each biomarker) were initially analyzed as continuous covariables for maximum efficiency; the estimated effects were expressed as odds ratios (ORs) of being frail vs. non-frail or prefrail vs. non-frail for each standard deviation unit increase in the log (of each biomarker), so that the relative contributions of each biomarker could be readily compared. In order to examine the role of the different inflammatory markers at baseline in the progression of frailty syndrome at 1 year-follow-up, we devised a predictive model for this follow-up outcome, including univariate analysis variables at a 5% significance level. In the longitudinal analysis, logistic regression was also used to examine associations between inflammatory biomarkers and main clinical variables relevant for frailty syndrome and PCa severity such as age, Gleason score, comorbidity index, presence or not of bone metastases and prostatectomy recorded at baseline and incident frailty progression at 1-year follow-up. The model building for all analyses included biomarkers and covariates of significance and relevance, with in which age, Gleason score, the presence of metastatic disease and prostatectomy, the comorbidity index were taken into account in the multivariable analyses. The hazard ratios (HRs), their 95% confidence intervals (CIs), and the statistical significance (*p*) of each predictive variable were estimated. The discrimination accuracy of the predictive model was calculated using C-statistics (area under the receiver operating characteristic curve; AUC). Statistical significance was set at a *p*-value of less than 0.05. All statistical analyses were performed using the SPSS software package (version 24.0; SPSS, Inc., Chicago, IL, USA).

## 5. Conclusions

In conclusion, in the present study, we have demonstrated that (1) more than half of patients with prostate cancer undergoing ADT progressed in the severity of their frailty syndrome; (2) cross-sectional studies performed in the same patients at baseline and 1 year later show that some inflammatory cells and soluble inflammatory markers in blood are associated with the severity of the frailty syndrome; and (3) a high Il-6 level and low lymphocyte count distinguishes patients whose frailty syndrome will progress over time. Taken together, these findings represent a first step toward the development of an optimized panel of biomarkers to complete the analysis of functional impairment in frail patients undergoing ADT, and help clinicians to take individualized decisions regarding frail patients with prostate cancer. 

## Figures and Tables

**Table 1:** Sociodemographic and clinical characteristics.

Category	Variation	*n*	%
**Marital status**	Married	34	87.2
	Widower	1	2.6
	Divorced	3	7.7
	Others	1	2.6
**Cohabitation status**	Single	4	10.3
	With partner	28	71.8
	With family	7	17.9
**Educational level**	Without studies	7	17.9
	Primary	17	43.6
	Secondary	9	23.1
	University	6	15.4
**Previous prostatectomy**	Yes	13	33.3
	No	26	66.7
**Metastases**	Yes	8	23.1
	No	31	76.9
**ADT treatment**	Leuprorelin	10	25.7
	Triptorelin	29	74.3
**NCCN risk score in PCa diagnoses**	Low	11	28.2
	Intermediate	8	20.5
	High	12	30.8
	Metastatic	8	20.5
**Gleason score**	5	2	5.1
	6	8	20.5
	7	17	43.6
	8	8	20.5
	9	4	10.3
**Ability to perform daily activities (Barthel index)**	90	6	15.4
	95	9	23.1
	100	24	61.5
**Charlson Comorbidity Index**	<4	22	59
	≥4	16	41

ADT = androgen deprivation therapy; NCCN = National Comprehensive Cancer Network.

**Table 2:** Inflammatory markers and frailty progression at follow-up.

Analytical Parameters at Baseline	Frailty Progression at Follow-Up (*N* = 23)		No Frailty Progression at Follow-Up (*N* = 16)		*p*
	Mean Value	±SD	Mean Value	±SD	
Lymphocytes count (10^3^/µL)	1.55	0.34	1.93	0.57	0.022
Neutrophils count ((10^3^/µL)	4.56	1.54	3.98	1.66	0.26
Monocytes count (10^3^/µL)	0.59	0.19	0.54	0.31	0.45
Eosinophil count (10^3^/µL)	0.21	0.14	0.18	0.09	0.41
Basophils count (10^3^/µL)	0.03	0.03	0.02	0.02	0.20
IL-6 (pg/mL)	2.12	0.55	1.78	0.45	0.038
IL-8 (pg/mL)	38.23	10.98	34.45	13.74	0.35
IL-beta (pg/mL)	0.25	0.10	0.29	0.09	0.19
TNF-α (pg/mL)	2.07	0.80	1.60	0.72	0.07
CRP (mg/L)	5.11	2.53	3.81	1.68	0.08
Age	72.70	9.31	70.88	10.78	0.58
Gleason score	7.17	1.07	7.00	0.97	0.60
Charlson Comorbidity Index	3.48	0.90	3.38	1.70	0.81
Percentage of patients with bone metastases	26.1%	-	18.8%	-	0.59
Percentage of patients underwent prostatectomy	60.9%	-	75.0%	-	0.36
